# Supplementary material for: Yiai Fuzheng decoction inhibits triple-negative breast cancer by remodeling the immune microenvironment
Source: Front Immunol. 2025 Sep 30;16:1615631. doi: 10.3389/fimmu.2025.1615631 (PMC12518410; doi:10.3389/fimmu.2025.1615631)
Supplement: Supplementary file 2 [file DataSheet1.zip › Supplementary File 2/Treatment/C_vs_H/07_Pathway_Analysis/Pathway_Analysis/Sig_Pathways_ID/Cysteine and methionine metabolism.pdf]

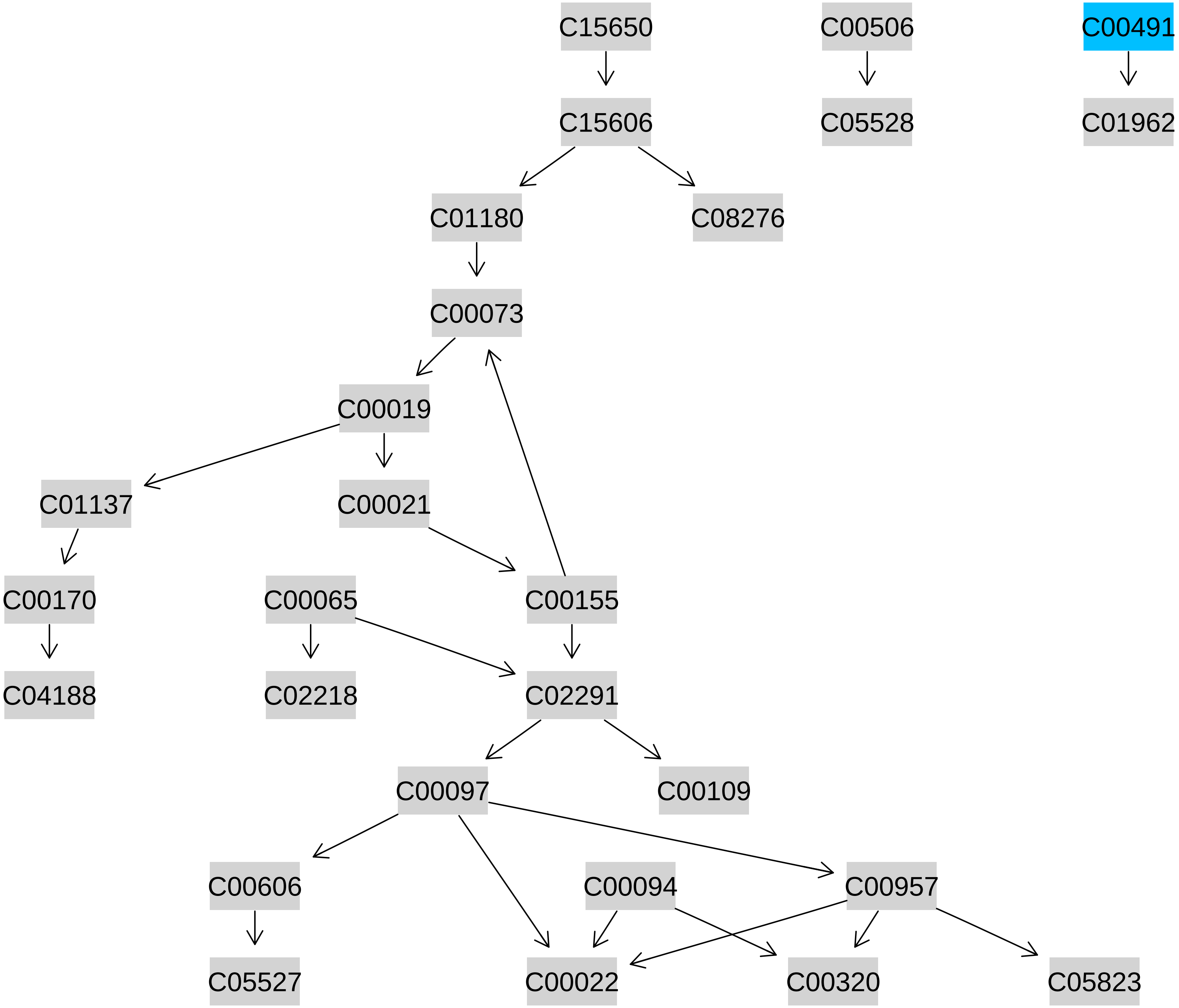
C15650
C00506
C00491
C15606
C05528
C01962
C01180
C08276
C00073
C00019
C01137
C00021
C00170
C00065
C00155
C04188
C02218
C02291
C00097
C00109
C00606
C00094
C00957
C05527
C00022
C00320
C05823
